# Surface charge-dependent mitochondrial response to similar intracellular nanoparticle contents at sublethal dosages

**DOI:** 10.1186/s12989-021-00429-8

**Published:** 2021-09-26

**Authors:** Xiaoting Jin, Haiyi Yu, Ze Zhang, Tenglong Cui, Qi Wu, Xiaolei Liu, Jie Gao, Xingchen Zhao, Jianbo Shi, Guangbo Qu, Guibin Jiang

**Affiliations:** 1 School of Public Health, Qingdao University, Qingdao, 266071 People’s Republic of China; 2 State Key Laboratory of Environmental Chemistry and Ecotoxicology, Research Center for Eco-Environmental Sciences, Chinese Academy of Sciences, Beijing, 100085 People’s Republic of China; 3 University of Chinese Academy of Sciences, Beijing, 100049 People’s Republic of China; 4 School of Environment, Hangzhou Institute for Advanced Study, UCAS, Hangzhou, 310000 People’s Republic of China

**Keywords:** Nanoparticle, Mitochondrial response, Surface property, Sublethal dosage, Intracellular content

## Abstract

**Background:**

Considering the inevitability for humans to be frequently exposed to nanoparticles (NPs), understanding the biosafety of NPs is important for rational usage. As an important part of the innate immune system, macrophages are widely distributed in vital tissues and are also a dominant cell type that engulfs particles. Mitochondria are one of the most sensitive organelles when macrophages are exposed to NPs. However, previous studies have mainly reported the mitochondrial response upon high-dose NP treatment. Herein, with gold nanoparticles (AuNPs) as a model, we investigated the mitochondrial alterations induced by NPs at a sublethal concentration.

**Results:**

At a similar internal exposure dose, different AuNPs showed distinct degrees of effects on mitochondrial alterations, including reduced tubular mitochondria, damaged mitochondria, increased reactive oxygen species, and decreased adenosine triphosphate. Cluster analysis, two-way ANOVA, and multiple linear regression suggested that the surface properties of AuNPs were the dominant determinants of the mitochondrial response. Based on the correlation analysis, the mitochondrial response was increased with the change in zeta potential from negative to positive. The alterations in mitochondrial respiratory chain proteins indicated that complex V was an indicator of the mitochondrial response to low-dose NPs.

**Conclusion:**

Our current study suggests potential hazards of modified AuNPs on mitochondria even under sublethal dose, indicates the possibility of surface modification in biocompatibility improvement, and provides a new way to better evaluation of nanomaterials biosafety.

**Supplementary Information:**

The online version contains supplementary material available at 10.1186/s12989-021-00429-8.

## Introduction

Nanoparticles (NPs) have been developed for multiple fields, such as textiles, cosmetics, electronics, biomedical applications, and environmental science and technology advances [1, 2]. Given that it is inevitable for humans to frequently contact NPs, understanding the environmental safety, and biological effects of NPs is important for their practical and rational usage [3, 4]. After the application of some NPs into the environmental compartments, NPs could be engulfed by environmental organisms and transferred to human body.

The biological effects of NPs are highly determined by their physicochemical properties [5, 6]. Among these properties, the diameter of NPs is an important factor influencing the interaction between NPs and biological systems [7]. Some NPs could be rapidly depredated in the ambient environment, which could exert side effects on not only environmental organism but also mammals [8]. The diameter of NPs greatly influences how and where NPs enter the cell, thus determining the toxicity of NPs [9]. For example, gold nanoparticles (AuNPs) with smaller diameter (3 and 6 nm) can enhance the production of proinflammatory cytokines and cause more cytotoxicity than 40 nm [10]. Another important property affecting the nano-bio interface is the surface of nanoparticle properties, which influence the interaction of NPs with biological system, which could lead to different side effects [3, 5, 11]. It has been reported that the content of internalized poly (ethylene glycol) (PEG)-NPs was less than that of mercaptoundecanoic acid (MUA)- or dodecyl amine-modified poly (isobutylene-alt-maleic anhydride) (PMA)-NPs and caused less cytotoxicity [12]. Our and other studies demonstrated that the surface of rare earth oxide NPs could interact with phosphorus of biological systems, which triggered the death of macrophages, the inflammation, and pulmonary injury [13, 14]. The properties of surface also determine the interaction of NP with soluble biological molecules such as proteins to form different types of protein corona complex, which also involves their biological effects [15–17].

Macrophages, as the dominant type of cell in the innate immune system, play a critical role in the monitoring and clearance of abnormally endogenous or exogenous substances in many tissues [18]. This immune cell has a vital role in protecting against attacks by invading exogenous particles, therefore becoming one of the most common cell types where NPs tend to accumulate [19, 20]. Therefore, upon exposure to NPs, macrophages, as the most sensitive cell type, mediate various side effects [21–23], including chronic inflammation and fat accumulation [24], pulmonary toxicity [25], and the promotion of tumor migration [26]. After being phagocytized by macrophages, NPs mainly accumulate in the lysosome, which could mediate the leakage of the lysosomal enzymes to trigger a pro-inflammatory immune response [27]. In addition to lysosomes, mitochondria are another sensitive organelle upon exposure to NPs, which can mediate oxidative stress and even cell death of macrophages [28].

Mitochondria, the energy factories of cells, can provide energy for macrophages through the mitochondrial respiratory chain and oxidative phosphorylation processes [29]. Mitochondrial signaling and metabolism are also involved in the activation and function of macrophages [30]. Moreover, the mitochondrion is an important mediator of cell homeostasis by regulating the stress response and metabolism [31]. Mitochondrial biogenesis is responsible for ROS generation and oxidative stress caused by NPs [32, 33]. Intracellular NPs can contribute to mitochondrial structural damage, respiratory chain dysfunction, and metabolic disorders and finally lead to an imbalance in cell energy [12, 32]. However, previous research on the effect of NPs on mitochondria has primarily served to explain the possible mechanism of cell death and cytotoxicity caused by high-dose NPs, whereas only a few studies have investigated the mitochondrial response to sublethal doses of NPs [13, 34]. Considering that the widespread application of NPs inevitably increases unwanted low-dose environmental exposure [35], studies on the environmental toxicity and health effects should be based on actual environmental exposure concentrations. At the low dosages that humans are usually exposed to, although NPs would not cause obvious cell death, these agents also showed the potential to interfere with cellular function [36]. For example, silver nanoparticles (AgNPs) did not cause obvious cell death at sublethal concentrations from 2 to 8 μg/mL but impaired the function of the mitochondrial respiratory chain and ATP production [34]. However, the effects on mitochondria in macrophages upon exposure to low dosages remain unclear.

Among numerous types of NPs, AuNPs show great promise and have been widely applied in medical and research fields, such as drug delivery, gene delivery, photothermal therapy, antibacterial therapy, and bioimaging [37]. Due to their unique optical, electronic, sensing, and biochemical characteristics, AuNPs were usually chosen to study the interaction with biological systems and crucial influencing factors [38]. It has been reported that AuNPs have an effect on mitochondrial structure and function. For example, mitochondria affected by 20 nm AuNPs at a concentration of 30 μg/mL were swollen in human placental pericytes [39]. Another research reported that 30 nm AuNPs cause alterations in mitochondrial mass and dysfunction in AuNPs-treated *C. albicans* cells [40]. Therefore, with different coatings and diameters, AuNPs were a suitable model to study the effect of AuNPs at sublethal concentrations on the mitochondrial response.

In this study, based on sublethal dosages in real exposure scenarios and with further consideration of the internal dose, the mitochondrial morphology, mitochondrial structure, ROS generation, ATP content, and expression of mitochondrial respiratory chain complexes in macrophages upon AuNP exposure with different coatings and diameters were investigated. Cluster analysis, two-way ANOVA, and multiple linear regression were performed to determine and compare the correlation of different properties and mitochondrial responses. Through the comparison, the sensitive indicator of the mitochondrial response to the sublethal dosage of AuNPs was discussed.

## Results and discussion

### Sublethal dosage for AuNPs exposure to macrophages

The properties of AuNPs with different diameters (5 nm or 50 nm) and coatings (BPEI, PVP, lipoic acid, tannic acid, citrate, and mPEG) were first determined. Additional file 1: Fig. S1A shows that all AuNPs were approximately spherical and had uniform diameters. The hydrodynamic diameters of AuNPs detected by DLS were slightly higher than the diameters obtained from TEM (Additional file 1: Fig. S1B), which may be caused by ligand adsorption and electrical double layers on the surface of the AuNPs. Most of the coated AuNPs were negative in both water and DMEM, wherein there was slightly more negative potential in water (Additional file 1: Fig. S1C). The surface charge of BPEI-AuNPs was almost positive in the two types of media, and only 5 nm BPEI-AuNPs were negatively charged in DMEM. This phenomenon was regarded as the formation of protein coronas on particles, which leads to a change in zeta potential [41].

**Fig. 1:**
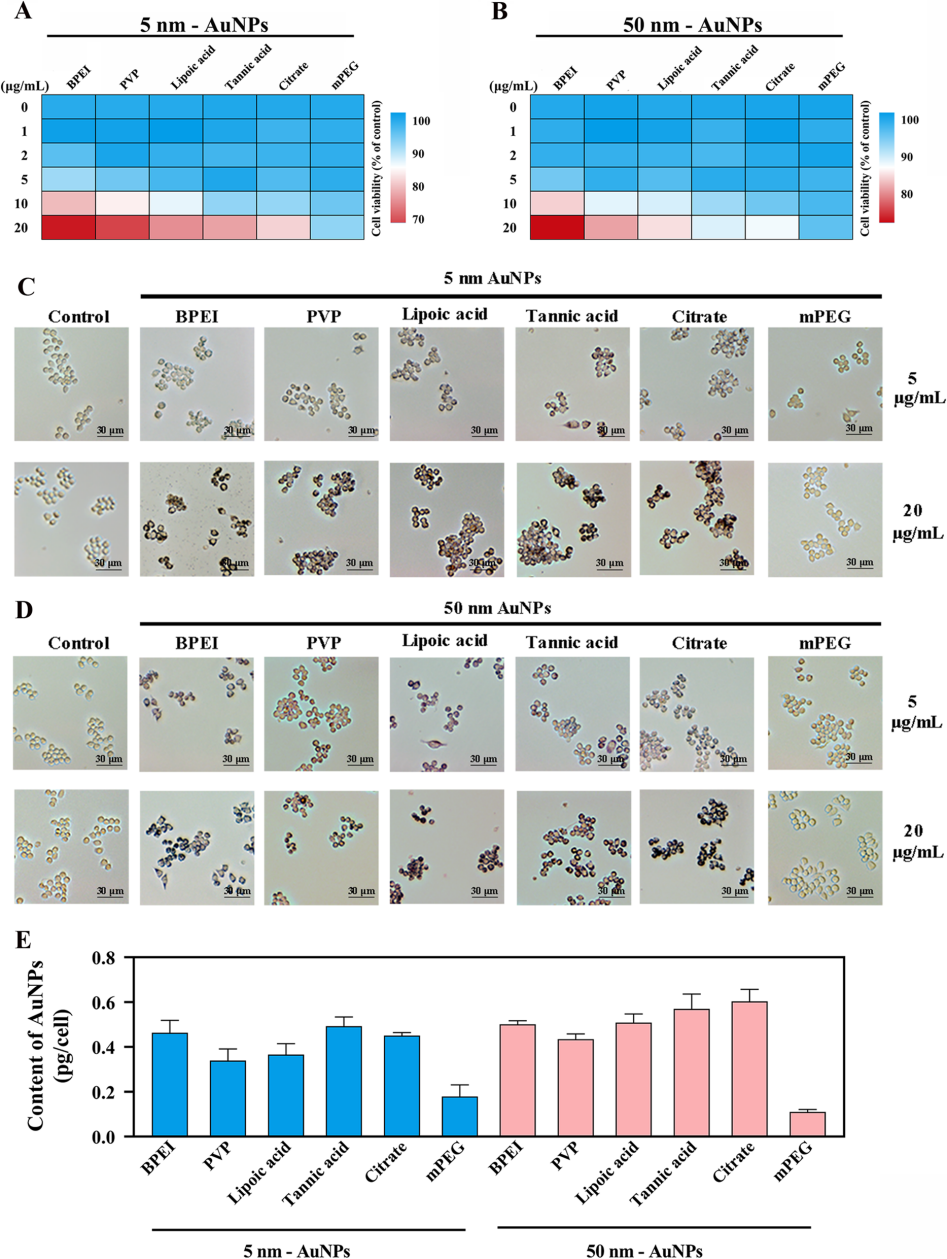
Determination of the sublethal dose and intracellular content of AuNP exposure on macrophages. Cell viability (n = 3) stimulated by a series of concentrations (0, 1, 2, 5, 10, and 20 μg/mL) of **A** 5 nm and **B** 50 nm AuNPs with different coatings for 24 h. Morphology of cells exposed to **C** 5 nm and **D** 50 nm AuNPs (5 and 20 μg/mL) with different coatings for 24 h. The scale bar is 30 μm. **E** The intracellular content of AuNPs (mean ± SD, n = 3) with different diameters and coatings analyzed by ICP-MS

To determine the noncytotoxic concentration, the cell viability of macrophages treated with a series of concentrations of AuNPs was assessed. As shown in Fig. 1A, B and Additional file 1: Fig. S2, none of the AuNPs showed an apparent alteration in cell viability when the concentration was at or lower than 5 µg/mL. At 20 µg/mL, except for mPEG-AuNPs, the cell morphologies were disturbed by AuNPs. At 5 µg/mL, there was no apparent morphological change upon treatment with AuNPs (Fig. 1C, D). Data from Annexin-V/PI dual staining further confirmed that AuNPs at 5 µg/mL did not cause dramatic cytotoxicity (Additional file 1: Fig. S3). The noncytotoxic concentration selected in the study was also included in the range of expected environmental concentrations (1.6 to 16.6 µg/mL) in vitro, which was based on the calculation model of the National Institute for Occupational Safety and Health [42]. Therefore, 5 µg/mL was considered the sublethal dose for RAW264.7 cells in the current study.

Many studies have indicated that the internal content of NPs is one of the pivotal factors influencing the interaction of NPs with organelles after exposure [5, 13]. ICP-MS was thus performed to evaluate the intracellular content of AuNPs. According to the calibration standard (Additional file 1: Fig. S4), at the same external exposure dose of 5 µg/mL, the intracellular 5 nm and 50 nm BPEI-, PVP-, lipoic acid-, tannic acid-, citrate-, and mPEG-AuNPs were 0.463, 0.339, 0.366, 0.493, 0.451, 0.179, 0.501, 0.435, 0.509, 0.570, 0.603 and 0.110 pg/cell, respectively. The 5 nm and 50 nm mPEG-AuNP contents were lower than half of those of the other five AuNPs, with averages of 0.179 and 0.110 pg/cell, respectively. A series of studies have demonstrated that the cellular uptake of PEG-coated AuNPs was less than that of other coated AuNPs; for example, SK-BR-3 breast cancer cells preferred to take up AuNPs in the following order: poly (allylamine hydrochloride), anti-HER2 antibody and PEG [43]. PEG is often used to reduce the uptake of NPs by macrophages.

However, except for mPEG-AuNPs, the other five types of AuNPs had little disparity (Fig. 1E). The similar intracellular content indicated that BPEI-, PVP-, lipoic acid-, tannic acid-, and citrate-coatings showed negligible effects on the cellular uptake of AuNPs. This fact might be attributed to the powerful phagocytic ability of macrophages toward NPs, which makes little difference in the effect of nanoparticles with different sizes on the endocytosis of macrophages [44]. However, other non-phagocytic cells, such as ovarian cancer cells, showed a different situation that the larger particles were internalized at a much higher amount compared to the smaller counterparts [45]. In addition, the low exposure concentration (5 μg/mL) may be another reason for the little difference in intracellular nanoparticle content. Therefore, to compare the effect of different AuNP properties on macrophage mitochondria under sublethal doses and exclude the influence of internal content (Fig. 1E), BPEI-, PVP-, lipoic acid-, tannic acid-, and citrate-AuNP were used for further research.

### Effects of AuNPs on the mitochondrial morphology and structure at sublethal dosages

**Fig. 2:**
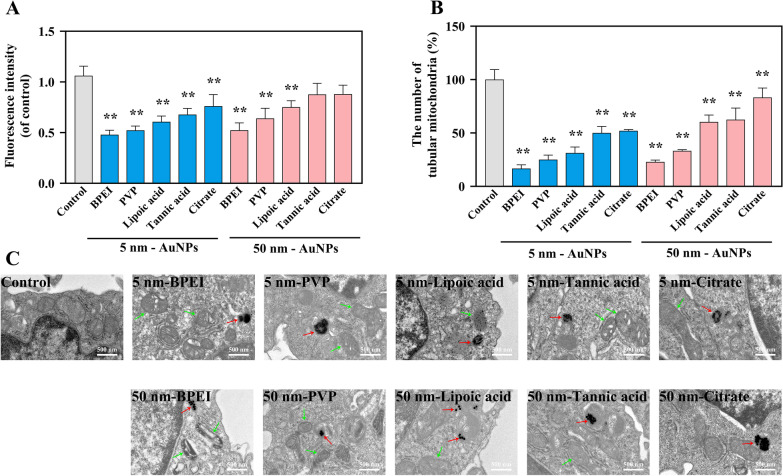
The alteration of mitochondrial morphology and structure in macrophages stimulated by different coated AuNPs (5 μg/mL) for 24 h. **A** The fluorescence intensity of mitochondria (mean ± SD, n = 3) using the MitoTracker Red assay. **B** The number of tubular mitochondria (mean ± SD, n = 3). *p < 0.05, **p < 0.01, compared with control. **C** Representative TEM images of macrophages. The scale bar is 500 nm. Red arrows point to the location of the internalized AuNPs, and green arrows point to the injured mitochondria

To evaluate the influence of AuNPs with different diameters and coatings, the mitochondrial morphology and structure of RAW264.7 cells were evaluated after exposure for 24 h. As shown in Additional file 1: Fig. S5, AuNPs altered the mitochondrial morphology to different degrees. Compared with the control, AuNPs exposure reduced the mitochondrial fluorescence intensity and the number of tubular mitochondria, both of which showed an analogous downward trend (Fig. 2A, B). Of note, BPEI-AuNP always caused the maximum effects. The representative TEM images further demonstrated alterations in mitochondrial morphology and structure (Fig. 2C). For both 5 nm and 50 nm NPs, BPEI-AuNP, PVP-AuNP, lipoic acid-AuNP, and tannic acid-AuNP displayed higher swelling, vacuolization, and cristae fracture, as indicated by the green arrows. Taken together, these results showed that AuNPs with different diameters and coatings at sublethal doses can cause damage to mitochondrial morphology and structure. Among all of the AuNPs, BPEI-AuNPs caused a maximal effect on mitochondria.

Previous studies have indicated the different toxic effects of AuNPs on mitochondria. Karataş et al. [46] found that AuNPs can form aggregates in the cytosol away from the mitochondria and did not cause substantial damage to mitochondria. However, in another study, AuNPs could be gradually trafficked to the mitochondria, where they reside in an aggregated state, making mitochondria somewhat swollen and round and causing mitochondrial crista to partially disappear and vacuolize [47]. Our study serves as a proof-of-concept that both 5 nm- and 50 nm-coated AuNPs agglomerated in the lysosomes of the cytoplasm but not mitochondria. However, the intracellular AuNPs also caused swelling, vacuolization, and round-shaped mitochondria.

### Impacts of AuNPs with different diameters and coatings on ROS generation and ATP content

**Fig. 3:**
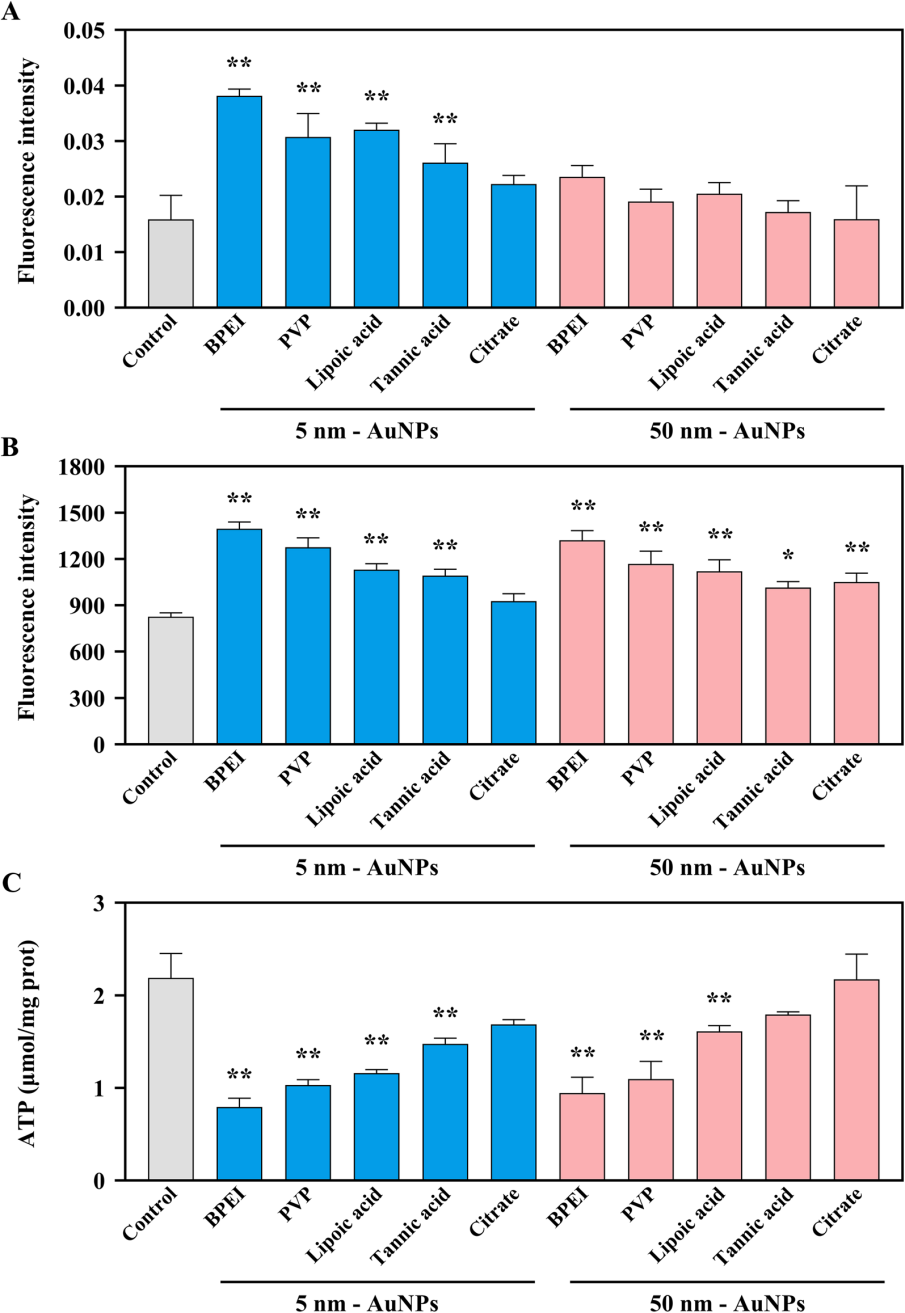
Total ROS, mitochondrial ROS, and ATP content after different coated AuNP (5 μg/mL) exposures for 24 h. **A** The levels of total ROS. **B** The levels of mitochondrial ROS. **C** The contents of ATP. *p < 0.05, **p < 0.01, compared with control. Data are presented as means ± SD (n = 3)

The mitochondria have a central role in ATP production and ROS generation, and intracellular levels of ATP and ROS can reflect mitochondrial function to some extent [31, 48]. Therefore, the total ROS, mitochondrial ROS, and ATP content upon treatment with AuNPs were evaluated. As shown in Additional file 1: Fig. S6, alterations in total ROS content were observed in AuNP-treated cells, and the degree of increased ROS was highly dependent on the diameter and coating. According to the quantitative results shown in Fig. 3A, the content of total ROS was significantly elevated (*p* < 0.01) after exposure to 5 nm BPEI-, PVP-, lipoic acid-, and tannic acid-AuNP. Furthermore, mitochondrial ROS was also increased (Additional file 1: Fig. S7), especially in cells stimulated with 5 nm and 50 nm BPEI-, PVP-, lipoic acid-, tannic acid-AuNP, and 50 nm citrate-AuNP (Fig. 3B, *p* < 0.01). Similarly, many studies have shown that NPs could induce ROS production [12, 49]. For example, exposure of pristine graphene at sublethal doses (5 µg/mL) for 48 h induced ROS generation in RAW264.7 cells [49]. Our results indicated that a low AuNP concentration could also cause an elevation of ROS in RAW264.7 cells. Moreover, NPs, including Au, Pt, TiO_2_, SiO_2,_ and Fe_2_O_3,_ increased the intracellular ROS of HUVECs at noncytotoxic concentrations, and the degree of change was closely related to the diameter and surface properties of the NPs [50]. In addition, AuNPs could deplete the intracellular antioxidant pool, stimulate ROS production, and cause oxidative stress, finally leading to cell necrosis and apoptosis [51, 52].

As shown in Fig. 3C, coated AuNP-treated cells showed significantly lower ATP content than untreated cells. ATP levels were reduced by 2.8, 2.1, 1.9, and 1.5 times after exposure to 5 nm BPEI-AuNPs, PVP-AuNPs, lipoic acid-AuNPs, and tannic acid-AuNPs for 24 h, respectively, compared with the control group (*p* < 0.01). The exposure of 50 nm BPEI-AuNPs, PVP-AuNPs, and lipoic acid-AuNPs also led to reduced ATP content (Fig. 3C, *p* < 0.01). Similar decreases in ATP after AuNP exposure have been presented in many previous studies, and the decrease was associated with cell cycle arrest [53, 54]. In the study of Yen et al. [10], AuNPs ranging from 2 to 40 nm significantly inhibited the proliferation of J774A.1 macrophages, which further demonstrates that AuNPs may affect ATP production by macrophages through cell cycle arrest. Moreover, changes in anabolic states were also associated with the activation of proinflammatory macrophages, such as enhanced glycolytic metabolism and inhibited mitochondrial oxidative phosphorylation in inflammatory macrophages [55]. The reduction in ATP caused by AuNPs suggests the inflammatory response of macrophages. Furthermore, the inhibition of ATP levels by AuNPs could be ascribed to the occurrence of apoptosis or necrosis, which is combined with changes in ROS levels [32]. Although there was no significant change in cell activity under the noncytotoxic dose, the changes in ROS and ATP at the subcellular level still indicated the perturbed normal physiology status of the cell, further suggesting the potential harm of sublethal dose AuNPs with different diameters and coatings.

### Correlation between mitochondrial alteration and AuNP properties

**Fig. 4:**
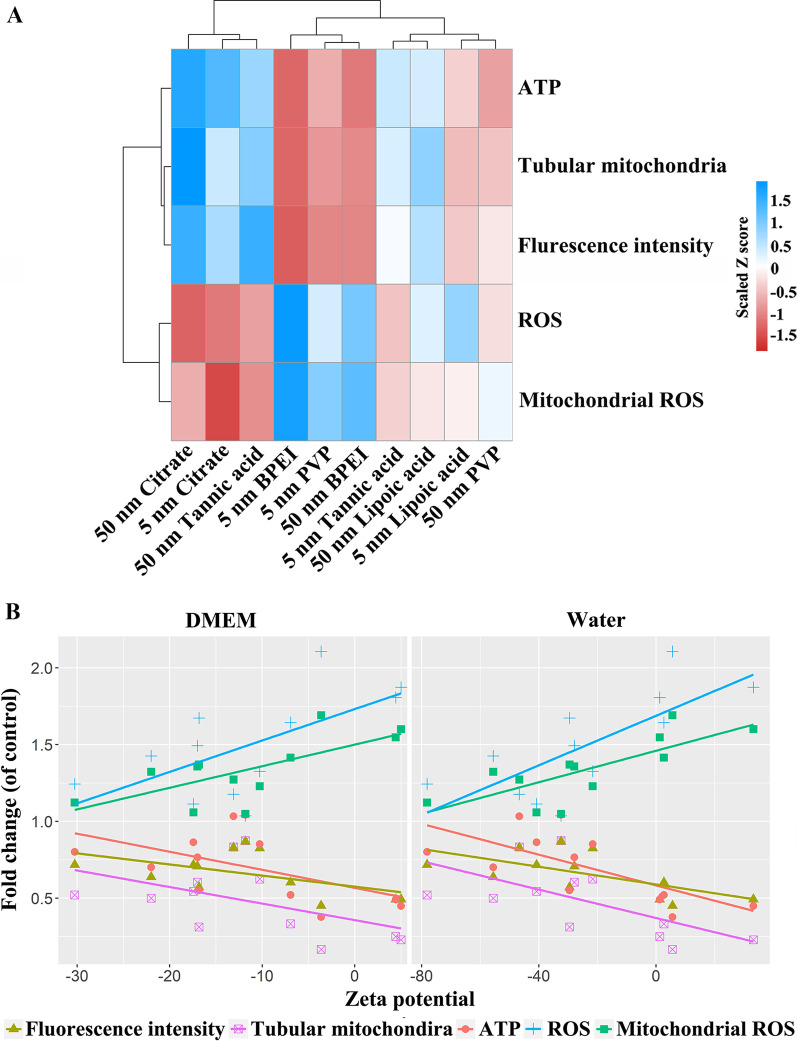
Correlation analysis of the mitochondrial response and AuNP properties. **A** Cluster analysis heatmap of the contribution from diameter and coating to the mitochondrial response. **B** Linear regression analysis between mitochondrial response and coating-related zeta potential changes. Fold change: the fold change of the mitochondrial response in DMEM or water

To determine the correlation of different properties and mitochondrial response and further elucidate the contribution of different properties to mitochondrial response at noncytotoxic dose AuNP exposure, cluster analysis, two-way ANOVA, and multiple linear regression were next performed. As shown in the heatmap of cluster analysis (Fig. 4A), the mitochondrial response was divided into two degrees. The alterations induced by 5 nm and 50 nm citrate-AuNP and 50 nm tannic acid-AuNP were classified into one degree, while those in the 5 nm and 50 nm BPEI-AuNP, PVP-AuNP, lipoic acid-AuNP, and 5 nm tannic acid-AuNP groups were classified into another degree. Surface properties led to the classification of two degrees, whereas the contribution of diameter was small. Further comparative analysis (shown in Additional file 2) illustrated that there were also differences between any two coatings with the same particle diameter for most of the mitochondrial indexes (*p* < 0.05). However, no obvious differences in most indicators were found between two diameters with the same coating. These results indicated that the surface properties exerted a higher impact on mitochondria in macrophages than the diameter, and final multiple linear regression (Additional file 1: Table S1) further proved this conclusion.

According to the result of different coated AuNP characteristics (Additional file 1: Fig. S1C), different coatings lead to an obvious change in zeta potential. Meanwhile, based on the result that the surface properties exerted a higher impact on mitochondria and the importance of zeta potential in surface properties, we further analyzed the correlation between zeta potential and mitochondrial response. The data from correlation analysis revealed that the mitochondrial response is related to the zeta potential (Additional file 1: Table S2). With increasing zeta potential, the mitochondrial fluorescence intensity, the number of tubular mitochondria, and the ATP level decreased. In comparison, total ROS and mitochondrial ROS were increased (Fig. 4B). These data suggested that the differences in mitochondrial responses caused by different coatings may largely be related to the coating-related zeta potential alteration, and the higher zeta potential had a stronger effect on the mitochondria, indicating that the mitochondrial response induced by the sublethal dose of AuNP exposure might be surface charge-dependent.

Previous studies have concluded that the interactions of NPs with biological systems are responsible for the execution of NP functions and eventual toxicity [5, 56]. Though diameter and coating both could affect the interaction, diameter mainly influenced the cellular uptake pathways through a variety of diameter-dependent interactions with the lipid bilayer, while coating-related surface properties affected the membrane interactions through many kinds of approaches. Different from those unstable NPs [57], AuNPs are the most stable NPs in the ambient environment and even within cells for a long time, which is a proper model to investigate the effects of surface on its toxicity. Among the coating-related surface properties, the zeta potential of nanoparticles is one of the key factors. Many studies have shown that cells are more effective at uptaking positively charged AuNPs than negatively charged and neutral AuNPs [5, 58]. The reason for this phenomenon is that the cell membrane is mostly negatively charged so that AuNPs with greater zeta potential can be tightly combined and internalized to a greater extent than AuNPs with less zeta potential due to electrostatic interactions [59]. Moreover, the membrane penetration ability of positively charged NPs was greater than that of neutral and negatively charged NPs, leading to a larger toxic response [43, 60]. As per the literature, the cellular uptake and subcellular localization of NPs greatly depended on their surface charge of polymeric coating. When NPs were internalized in the lysosomes, the acidic environment of lysosomes activated oxidase in NPs to induce toxicity, while lower toxicity was found in the cytoplasm of cells [61]. However, our results showed that there was little difference in the intracellular contents of AuNPs with different coatings except for mPEG-AuNPs.

### The response of mitochondrial respiratory chain complexes and macrophage function induced by typical AuNPs

**Fig. 5:**
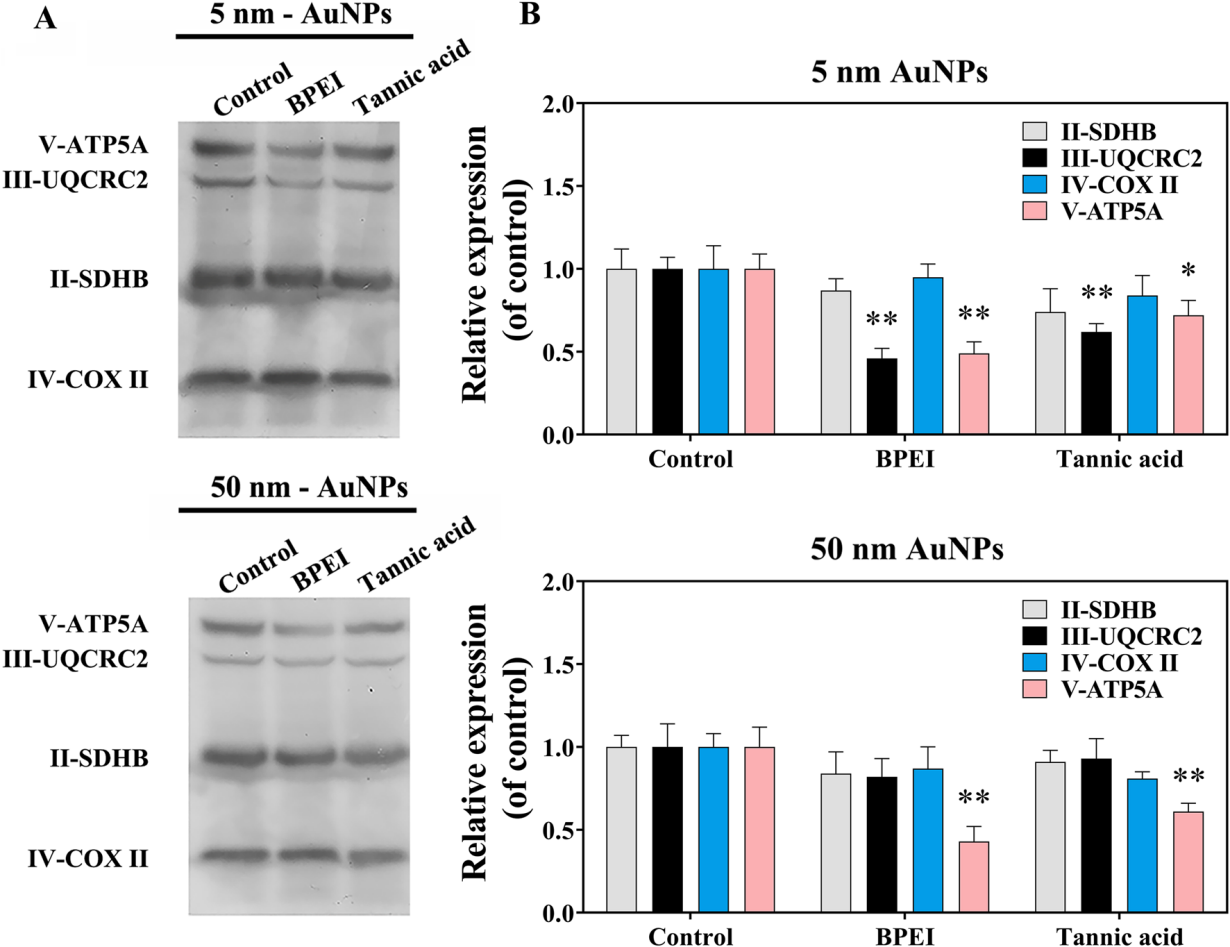
The expression of mitochondrial respiratory complexes and phagocytic ability of RAW 264.7 after exposure to 5 nm and 50 nm BPEI- and tannic acid-AuNPs for 24 h. **A** Representative lanes of mitochondrial respiratory chain complex II–V (n = 3 blots in total) using western blot. **B** The semi-quantitative results of mitochondrial respiratory chain complexes expression (n = 3) by gray scanning. *p < 0.05, **p < 0.01, compared with control

Considering the obvious changes in ATP and ROS disturbed by AuNPs, two different coated AuNPs (i.e., BPEI-AuNPs and tannic acid-AuNPs) were selected to further study the alteration of the respiratory electron transport chain, a key site for ATP and ROS generation. As shown in Fig. 5A, B, there was no observable alteration in complex II (SDHB) or complex IV (COX II) in macrophages stimulated by BPEI-AuNPs and tannic acid-AuNPs at both 5 nm and 50 nm. The expression of complex III (UQCRC2) was downregulated in the 5 nm AuNP-exposed groups but not in the 50 nm AuNP groups when compared with the control group. However, there was a conspicuous decrease in complex V (ATP5A) in macrophages treated with both 5 nm and 50 nm AuNPs. The activities of complex III and complex V were further determined. Interestingly, 5 nm and 50 nm BPEI- and tannic acid-AuNPs had no effect on the activity of complex III in both RAW264.7 and J774A.1 macrophages (Additional file 1: Fig. S8A, C). However, a decreased activity of mitochondrial complex V in the 5 nm AuNP-exposed groups was observed in both RAW264.7 and J774A.1 macrophages (Additional file 1: Fig. S8B, D), which was consistent with the results of western blots (Fig. 5A, B). The above data suggested that complex V of the mitochondrial respiratory chain was more sensitive to the exposure of AuNPs and can be listed as a meaningful biomarker for the high-throughput screening of NP-induced mitochondrial dysfunction at sublethal doses.

Some studies have indicated that NP exposure could cause changes in the mitochondrial respiratory chain [62, 63]. For example, NP exposure at a dose of 100 μg/mL downregulated the expression of mitochondrial respiratory chain complexes I, IV, and V in human bronchial epithelial (HBE) cells [62]. However, research on the effects of NP exposure on the respiratory chain has mainly focused on high doses. Studies on the effects of NP exposure at sublethal doses on mitochondrial respiratory chains are lacking. One study indicated that at sublethal concentrations, the expression of complexes I, IV, and V was downregulated after NP exposure for 24 h [64]. Therefore, the results of our study were supplements to the data on the effects of NPs on mitochondrial respiratory chains at sublethal doses. In a series of mitochondrial respiratory chain complexes, complex III is one of the pivotal points in ROS generation, and complex V is the key enzyme that phosphorylates ADP to ATP. The downregulation of complex III and V might result in a decrease in ROS and ATP. In our study, the content of ATP was decreased after exposure to AuNPs with different coatings and diameters (Fig. 3C), which is consistent with the downregulation of complex V. Therefore, it was presumed that AuNPs downregulated complex V and further led to a decrease in ATP generation. Similarly, studies have indicated that NP exposure inhibited the expression of complex V and impaired ATP production, consistent with the results of our study [62, 64]. However, ROS generation was increased after exposure to different AuNPs (Fig. 3B), which contradicted the downregulation of complex III. The reason for this contradiction might be the other sources of ROS, such as complex I, NADPH oxidases, xanthine oxidase, and nitric oxide synthase [65]. AuNPs may increase ROS production by influencing other sources of ROS. Alternatively, the increase in ROS might be related to the sensitivity of the methods used to detect mitochondrial ROS. Taken together, complex V might serve as a sensitive biomarker to indicate the effect on mitochondria under a low dose of NP exposure.

Subsequently, we examined the effects of AuNPs treatment on the biological functions of macrophages, such as the secretion of pro-inflammatory cytokines and phagocytic capacity of macrophages. The level of IL-6, a representative pro-inflammatory cytokine of macrophages [66], was significant increased in macrophages treated with 5 nm and 50 nm AuNPs when compared with the control group (Additional file 1: Fig. S9). However, there was no difference in the ability of phagocytosis between 5 and 50 nm BEPI-, tannic acid-AuNPs treated groups and control group in both RAW264.7 macrophages and J774A.1 macrophages (Additional file 1: Fig. S10). Similarly, a prior study by Chen et al. [32] has reported that noncytotoxic dose of TiO_2_NPs treatment had no effects on phagocytic capability of RAW264.7 cells, but cytotoxic dose produced attenuation on that. Moreover, the TiO_2_NPs caused mitochondrial dysfunction and activated inflammatory responses under both the cytotoxic and noncytotoxic dose [32]. Overall, these results indicated that AuNPs with different coating and size at a similar internal exposure dose under sublethal concentration led to the IL-6 mediated inflammation response, which might be linked to the changes in mitochondrial morphology, structure, and function.

## Conclusion

Our current study suggested that AuNP exposure at a sublethal dose could contribute to damage to mitochondrial morphology, structure, and function, providing a deeper understanding of mitochondrial alterations upon exposure to AuNPs (Additional file 1: Fig. S11). Our findings evaluated the different coating and particle size of nanoparticles on mitochondrial alterations in the real environment upon the sublethal dose and the similar internal dose exposure. Except for mPEG-AuNPs, all of the other AuNPs selected showed similar intracellular contents. Therefore, even at similar intracellular contents, the mitochondrial response still showed a surface charge-dependent trend. The AuNPs with higher zeta potential led to the strongest effect on mitochondria. Our results also suggested that some relevant indicators of mitochondrial response, including ROS generation, ATP production, and the expression of complex V, can be used as sensitive indicators for the high-throughput screening of toxic NPs at low-dose exposure. However, because intracellular AuNPs did not directly contact or accumulate in mitochondria, the mechanisms responsible for the incurred charge-dependent mitochondrial response must be further explored. Energy metabolism plays an important role in the immune function of macrophages [55], and whether the changes in mitochondria caused by AuNPs will impact the function of macrophages warrants further evaluation.

## Material and methods

### Materials

The 5 nm and 50 nm AuNPs (1 mg/mL) in distilled water were commercially purchased from Nanocomposix Company (San Diego, CA, USA), and quality guarantee periods were 1 year when stored at 4 °C in darkness. The surfaces of 5 nm and 50 nm AuNPs were coated with branched polyethylenimine (BPEI), polyvinyl pyrrolidone (PVP), lipoic acid, tannic acid, sodium citrate, or polyethylene glycol monomethyl (mPEG). The working suspension was freshly prepared by diluting the sonicated stock solution with distilled water and thoroughly mixing before usage. The NPs remained monodispersed during the whole study, and no apparent aggregation was observed.

### Cell culture

RAW264.7 cells, a mouse monocytic/macrophage-like cell line, were obtained from the Institute of Biochemistry and Cell Biology (SIBS, CAS, Shanghai, China). The cells were cultured at 37 °C in 5% CO_2_ and 95% humidified air in DMEM/high glucose medium (HyClone, South Logan, UT, USA) with 10% fetal bovine serum (FBS, Boster, Pleasanton, CA, USA) and 1% penicillin (100 U/mL)/streptomycin (Solarbio, Beijing, China).

### Cell viability assay

RAW264.7 cells were previously seeded in 96-well plates and allowed to adhere overnight. Following stimulation with different coatings of 5 nm and 50 nm AuNPs (0, 1, 2, 5, 10, and 20 μg/mL) for 24 h, the medium was then replaced by 100 μL of fresh cell culture medium containing 10 μL of cell counting kit-8 (CCK-8, Boster, Pleasanton, CA, USA). After further incubation for 2 h at 37 °C, the absorbance at 450 nm was assessed using a microplate reader (Thermo Fisher Scientific, Waltham, MA, USA). The experiment was conducted in triplicate, and the relative viability of cells was determined as follows: cell viability (% of control) = (absorbance of treated sample/absorbance of control sample) × 100.

### Cellular morphology observation

For the observation of cellular morphology, RAW264.7 cells were exposed to 5 nm and 50 nm AuNPs (5 and 20 μg/mL) for 24 h with different coatings (BPEI, PVP, lipoic acid, tannic acid, citrate, and mPEG). The images were captured by an Eclipse TS100 inverted research microscope (Nikon, Tokyo, Japan).

### ICP-MS analysis

To determine the cellular uptake of AuNPs, cells were incubated with different coatings of 5 nm and 50 nm AuNPs (5 μg/mL) for 24 h, and the amounts of internalized AuNPs were measured with inductively coupled plasma-mass spectrometry (ICP-MS) analysis. After the treatment, cells were washed with ice-cold PBS three times and collected using 0.25% trypsin. Subsequently, cell numbers were counted using a hemocytometer, and cells were lysed with RIPA buffer (Solarbio, Beijing, China) at 4 °C for 1 h. Following quantitative analysis of lysates by a BCA kit (Thermo Fisher Scientific, Waltham, MA, USA), the lysates were then added to 100 μL of aqua regia (concentrated HNO_3_ (GR, 65.0%, Millipore, Billerica, MA, USA) and HCl (GR, 30%, Sinopharm Chemical Reagent Co., Ltd, Shanghai, China)) with a volume ratio of 1:3 for sample digestion at 60 °C until the solutions became transparent. The digested solutions were cooled to room temperature and diluted with 2% nitric acid solution (HNO_3_) to reach a final volume of 1 mL. Finally, the samples were subjected to ICP-MS (Agilent, Tokyo, Japan) to determine the AuNP contents. The gold stock solution (GSB 04-1715-2004, 1000 μg/mL in 1.5 mol/L HCl) was used as a calibration standard.

### Evaluation of mitochondrial morphology

RAW264.7 cells at a density of 100,000 cells/well on 12-well glass slides were stimulated with different coated 5 nm and 50 nm AuNPs (5 μg/mL) for 24 h. After the treatment, the mitochondrial morphology was analyzed using MitoTracker Red (Invitrogen, Carlsbad, CA, USA) as described in our previous study [67]. To holistically reflect the mitochondrial morphology, a quantitative analysis of tubular mitochondrial number in 100 cells per sample was performed, and the fluorescence intensity of MitoTracker Red was analyzed using ImageJ software (Version 1.8.0) to represent the alteration of mitochondrial number.

### Observation of mitochondrial structure using TEM

RAW264.7 cells were fixed with 2.5% glutaraldehyde for 2 h. Following postfixation with 1% osmium tetroxide (Sigma, St. Louis, MO, USA) for 1 h at 4 °C, the cells were rinsed several times with distilled water and dehydrated in a series of ethanol solutions (50, 70, 80, 90, and 100%). The dehydrated samples were embedded in epoxy resin, sliced, and stained with 2% uranyl acetate (KEYI Technology Development Ltd, Beijing, China) and 3% lead citrate (KEYI Technology Development Ltd, Beijing, China). Afterward, the cell morphology and representative TEM images were observed using a JEM 2100EX microscope (JEOL, Tokyo, Japan) under the help of the commercial-available technique service (Beijing ZKBC Technology Service Company Ltd, Beijing, China).

### ROS production assay

The content of intracellular reactive oxygen species (ROS) was detected using a 2′,7′-dichlorodihydrofluorescein diacetate (DCFH-DA, Beyotime, Shanghai, China) assay. Briefly, the cells were seeded in a 12-well plate at a density of 300,000 cells/well. After treatment with different AuNPs (5 μg/mL) for 24 h, the cells were incubated in 300 μL of 10 μM DCFH-DA at 37 °C for 30 min. After washing with PBS three times, cells were captured by a fluorescence microscope (IX73, Olympus, Japan). All the samples were photographed with the fixed microscopic parameters to ensure reliable results. To quantify the intracellular fluorescence, the fluorescent intensities and cell area were analyzed using ImageJ software. And the fluorescence intensity per unit area under each group was calculated.

### Mitochondrial ROS production

Levels of mitochondrial ROS were determined using the MitoSOX Red (M36008, Invitrogen, Carlsbad, CA, USA) assay. After treatment with 5 nm and 50 nm AuNPs (5 μg/mL) for 24 h, the cells were harvested by trypsinization, washed, and treated with 5 μM MitoSOX Red solution in Hank’s Balanced Salt Solution (HBSS, Gibco, Carlsbad, CA, USA) for 10 min at 37 °C. The cells were then resuspended in 500 μL of fresh HBSS and analyzed by flow cytometry (Novocyte 1040, ACEA Biosciences, San Diego, CA, USA).

### Analysis of intracellular ATP content

Cells were harvested following incubation with different coated 5 nm and 50 nm AuNPs (5 μg/mL) for 24 h. The intracellular ATP content was quantified by a commercial kit according to the manufacturer's instructions (Nanjing Jiancheng Biological Product, Nanjing, China), as described in our previous study [68]. The absorbance value was measured using a fluorescence microplate reader (Thermo Fisher Scientific, Waltham, MA, USA) at 636 nm. The level of ATP was expressed as μmol/mg protein (i.e., μmol/mg prot).

### Cluster analysis

Cluster analysis was performed using R 4.0.0 (R Foundation for Statistical Computing, Vienna, Austria) and RStudio. The variation multiples of each experimental group relative to the control group were calculated, and the data were scaled using the Z-score standardization method. The R pheatmap package and longest distance method were used for cluster analysis and cluster heatmap drawing.

### Correlation analysis between mitochondrial response and zeta potential

With the zeta potential as the independent variable and fold change compared to control as the dependent variable, the linear regression coefficient value was calculated using R 4.0.0 (R Foundation for Statistical Computing, Vienna, Austria) to clarify the relationship between the mitochondrial response and zeta potential in different NPs exposure groups. The Ggplot2 package was used to plot the figure.

### Determination of mitochondrial respiratory chain complexes

After stimulation with different coatings of 5 nm and 50 nm AuNPs (5 μg/mL) for 24 h, mitochondria in harvested cells were first obtained with a commercial kit following the manufacturer's protocol (Nanjing Jiancheng Biological Product, Nanjing, China). The mitochondrial proteins were then extracted using RIPA buffer lysis (Solarbio, Beijing, China). Following centrifugation at 10,000 rpm and 4 °C for 30 min, the concentrations of protein in the lysate were quantified by the BCA method (Thermo Scientific, Waltham, MA, USA). Mitochondrial extracted proteins were resolved by SDS-PAGE, transferred onto nitrocellulose membranes, and detected as described previously [69]. The total OXPHOS antibody cocktail (1:1000, ab110411, Abcam, Cambridge, MA, USA) and HRP-conjugated anti-mouse IgG (1:500, EasyBio, Beijing, China) were used as the primary antibody and secondary antibody, respectively.

### Statistical analysis

Statistical analyses were performed using SPSS 17.0 (IBM Corporation, Armonk, NY, USA) or GraphPad 8.0 (GraphPad Software, La Jolla, CA, USA). The results are expressed as the mean ± standard error (SE). The double factor variance analysis (two-way ANOVA) procedure was used to compare the significant differences between various groups, and the Tukey method was used to finish the multiple comparisons. For the *p* values, *p < 0.05, **p < 0.01 were marked as statistically significant and highly statistically significant, respectively.

## Supplementary Information

**Additional file 1: Fig. S1.** Morphology, diameter, hydrodynamic diameters, and zeta potentials of differently coated AuNPs. **Fig. S2.** Cell viability stimulated by a series of concentrations (0, 1, 2, 5, 10, and 20 μg/mL) of (A) 5 nm and (B) 50 nm AuNPs with different coatings (BPEI, PVP, lipoic acid, tannic acid, citrate, and mPEG) for 24 h. **Fig. S3.** Cell apoptosis stimulated by 5 nm and 50 nm AuNPs (5 μg/mL) with different coatings (BPEI, PVP, lipoic acid, tannic acid, citrate, and mPEG) for 24 h. **Fig. S4.** The standard curve and regression equation of gold. **Fig. S5.** The fluorescence images of mitochondrial morphology with Mito Tracker Red staining after different coated AuNPs (5 μg/mL) exposure for 24 h. **Fig. S6.** Fluorescence images of total ROS generation after exposure of 5 nm and 50 nm different coated AuNPs (5 μg/mL) for 24 h. **Fig. S7.** The mitochondrial ROS levels in macrophages stimulated by 5 nm and 50 nm different coated AuNPs (5 μg/mL) for 24 h. **Fig. S8.** The activities of complex III and complex V in macrophage upon BPEI-AuNPs and tannic acid-AuNPs treatment. **Fig. S9.** The content of IL-6 in RAW264.7 cells treated with 5 nm and 50 nm BPEI- and tannic acid-AuNPs (5 μg/mL) for 24 h. **Fig. S10.** The phagocytic capacity of macrophages after 5 nm and 50 nm BPEI- and tannic acid-AuNPs exposure. **Fig. S11.** Schematic diagram for surface charge-dependent mitochondrial response to similar intracellular nanoparticle contents at sublethal dosages. **Table S1.** Multiple linear regression on the contribution of diameter and coating to the mitochondrial response. **Table S2.** Correlation analysis of zeta potential and different mitochondrial responses.

**Additional file 2.** Results of two-way ANOVA (Excel Worksheets 1–2).

## Data Availability

All data analyzed within this study are included either in the manuscript or in the Additional File 1 and Additional File 2.

## Abbreviations

NPs: Nanoparticles
AuNPs: Gold nanoparticles
ROS: Reactive oxygen species
ATP: Adenosine triphosphate
PEG: Poly (ethylene glycol)
MUA: Mercaptoundecanoic acid
PMA: Poly (isobutylene-alt-maleic anhydride)
AgNPs: Silver nanoparticles
BPEI: Branched polyethylenimine
PVP: Polyvinyl pyrrolidone
mPEG: Polyethylene glycol monomethyl
SIBS: Institute of Biochemistry and Cell Biology
FBS: Fetal bovine serum
CCK-8: Cell counting kit-8
ICP-MS: Inductively coupled plasma-mass spectrometry
DCFH-DA: 2′,7′-Dichlorodihydrofluorescein diacetate
