# Supplementary material for: Surface charge-dependent mitochondrial response to similar intracellular nanoparticle contents at sublethal dosages
Source: Part Fibre Toxicol. 2021 Sep 26;18:36. doi: 10.1186/s12989-021-00429-8 (PMC8474914; doi:10.1186/s12989-021-00429-8)
Supplement: Supplementary file 1 — Additional file 1: Fig. S1. Morphology, diameter, hydrodynamic diameters, and zeta potentials of differently coated AuNPs. Fig. S2. Cell viability stimulated by a series of concentrations (0, 1, 2, 5, 10, and 20 μg/mL) of (A) 5 nm and (B) 50 nm AuNPs with different coatings (BPEI, PVP, lipoic acid, tannic acid, citrate, and mPEG) for 24 h. Fig. S3. Cell apoptosis stimulated by 5 nm and 50 nm AuNPs (5 μg/mL) with different coatings (BPEI, PVP, lipoic acid, tannic acid, citrate, and mPEG) for 24 h. Fig. S4. The standard curve and regression equation of gold. Fig. S5. The fluorescence images of mitochondrial morphology with Mito Tracker Red staining after different coated AuNPs (5 μg/mL) exposure for 24 h. Fig. S6. Fluorescence images of total ROS generation after exposure of 5 nm and 50 nm different coated AuNPs (5 μg/mL) for 24 h. Fig. S7. The mitochondrial ROS levels in macrophages stimulated by 5 nm and 50 nm different coated AuNPs (5 μg/mL) for 24 h. Fig. S8. The activities of complex III and complex V in macrophage upon BPEI-AuNPs and tannic acid-AuNPs treatment. Fig. S9. The content of IL-6 in RAW264.7 cells treated with 5 nm and 50 nm BPEI- and tannic acid-AuNPs (5 μg/mL) for 24 h. Fig. S10. The phagocytic capacity of macrophages after 5 nm and 50 nm BPEI- and tannic acid-AuNPs exposure. Fig. S11. Schematic diagram for surface charge-dependent mitochondrial response to similar intracellular nanoparticle contents at sublethal dosages. Table S1. Multiple linear regression on the contribution of diameter and coating to the mitochondrial response. Table S2. Correlation analysis of zeta potential and different mitochondrial responses. [file 12989_2021_429_MOESM1_ESM.docx]

**Supporting Information for**

**Surface charge-dependent mitochondrial response to similar intracellular nanoparticle contents at sublethal dosages**

Xiaoting Jin^1^, Haiyi Yu^1^, Ze Zhang^1^, Tenglong Cui^1^, Qi Wu^2^, Xiaolei Liu^2^, Jie Gao^2^, Xingchen Zhao^2^, Jianbo Shi^2,3,4^, Guangbo Qu^2,3,4*^, and Guibin Jiang^2,3,4^

^1^School of Public Health, Qingdao University, Qingdao 266071, P. R. China.

^2^State Key Laboratory of Environmental Chemistry and Ecotoxicology, Research Center for Eco-Environmental Sciences, Chinese Academy of Sciences, Beijing 100085, P. R. China.

^3^University of Chinese Academy of Sciences, Beijing 100049, P. R. China.

^4^School of Environment, Hangzhou Institute for Advanced Study, UCAS, Hangzhou 310000, P. R. China.

* Correspondence: gbqu@rcees.ac.cn

State Key Laboratory of Environmental Chemistry and Ecotoxicology, Research Center for Eco-Environmental Sciences, Chinese Academy of Sciences, Beijing 100085, P. R. China.

### Additional file 1

### Materials and methods

**Morphologies and particle diameters**

AuNP dispersions were loaded onto a 230-mesh perforated carbon foil supported by a copper grid and samples morphologies were observed using a JEOL 2100F high-resolution transmission electron microscope (TEM, JEOL, Japan) operating at 200 kV.

**Hydrodynamic diameter and zeta potentials**

Dynamic light scattering (DLS) measurements were conducted to determine the hydrodynamic radius and zeta potentials of AuNPs in deionized water and medium supplemented with 10% fetal bovine serum (FBS) using the Zetasizer Nano ZS (Malvern, UK) at room temperature.

**Annexin V-FITC/PI staining**

After being stimulated by 5 nm and 50 nm AuNPs (0, 5 μg/mL) for 24 h with different coatings (BPEI, PVP, lipoic acid, tannic acid, citrate, and mPEG), cells were harvested through the trypsinization and washed twice with PBS. The cells were centrifuged at 3000 rpm for 5 min to discard the supernatant. At least 100,000 cells from each sample were resuspended in 100 μL of binding buffer and incubated with 5 μL of FITC Annexin V and 2 μL of propidium iodide (PI; US Everbright, Jiangsu, China) for 15 min at 4 ℃. Subsequently, 400 μL of binding buffer was added prior to the measurement using a Flow Cytometer (Becton Dickinson, USA) with Cell Quest software (Becton Dickinson).

**The observation of cellular ROS**

2’,7’-dichlorodihydrofluorescein diacetate (DCFH-DA, Beyotime, China) was used to observe the cellular ROS. In brief, cells at the density of 100,000 cells/well were cultured on poly-L-lysine coated glass coverslips in 12 well plates in DMEM medium. After being exposed to different coated 5 nm and 50 nm AuNPs (5 μg/mL) for 24 h, the cells were fed with serum-free DMEM, loaded with 300 μL of DCFH-DA (10 μM) in the dark and kept in a CO_2_ incubator at 37℃ for 1 h. Uncombined DCFH-DA was removed by gently washing with PBS three times. Images were monitored in green fluorescence and bright-field channels using fluorescence microscopy (Delta Vision, USA).

**J774A.1 cell culture**

J774A.1 macrophages, another common murine macrophage cell line, were cultured in DMEM/high glucose medium (HyClone, South Logan, UT, USA) containing 10% FBS (Boster, Pleasanton, CA, USA) and 1% penicillin (100 U/mL)/streptomycin (Solarbio, Beijing, China) in a humidified atmosphere of 5% CO_2_ at 37°C.

**Measurement of mitochondrial respiratory chain complex III and V activities**

The activities of mitochondrial complex III and V in the RAW264.7 cells or J774A.1 cells were determined using commercial kits (Solarbio, Beijing, China). Briefly, the cells were seeded in 6-well plates, and exposed to 5 and 50 nm BEPI-, tannic acid-AuNPs (5 μg/mL) for 24 h after growing to 80% confluence. All assays were performed according to the manufacturer’s instructions. The activity of complex III in cell lysis was assayed by monitoring the increase in reductive cytochrome C with the changes in absorption at 550 nm recorded for 2 min using a microplate reader (Thermo Fisher Scientific, Waltham, MA, USA). The activity of complex V was measured with a determination of absorption at 660 nm recorded for 30 min using a microplate reader (Thermo Fisher Scientific, Waltham, MA, USA) through testing the increase in inorganic phosphorus. The enzyme activity displayed as U/mg prot (i.e. U/mg protein).

**Cell phagocytosis assay on macrophages**

The macrophages of J774A.1 and RAW264.7 were implanted in 12 well plates, and exposed to 5 and 50 nm BEPI-, tannic acid-AuNPs (5 μg/mL) for 24 h after reaching 80% confluence, respectively. Then, the culture medium was replaced by DMEM contained the yellow fluorescent-conjugated amine-modified polystyrene latex beads with a mean particle size of 1 μm (1:1000, Sigma, St. Louis, MO, USA), which has been pretreated in FBS for 30 min at 37 ℃, and was incubated with cells in the dark for 1 h according to the previously reported method.[1] The cells were subsequently collected with cell scraping, washed twice with ice-cold PBS and then resuspended with 200 μL PBS followed by flow cytometer (Beckman Coulter, Brea, CA, USA) to collect the number and mean fluorescence intensity of cells phagocytic yellow latex beads.

**Enzyme-linked immunosorbent assay for IL-6**

The level of IL-6 in cell lysis was measured using an enzyme-linked immunosorbent assay (ELISA) according to the manufacturer’s instructions (EIAab, Wuhan, China). Briefly, diluted culture supernatant was added to the pre-blocked plates. After a 2 h incubation at 37 °C, the fluid was removed and biotinylated anti-IL-6 antibody was added to the plates with a 1 h incubation at 37 °C. After incubated with streptavidin-HRP for 1 h at 37 °C, tetramethylbenzidine was added and incubated as the substrate reagent. The mixture was incubated at 37 °C in darkness for 15 min, and then the reaction was terminated by a stop solution. Absorbance was determined at a wavelength of 450 nm using a microplate reader (Thermo Fisher Scientific, Waltham, MA, USA).





**Fig. S1.** Morphology, diameter, hydrodynamic diameters, and zeta potentials of differently coated AuNPs. (A) TEM images of 5 nm and 50 nm AuNPs with different coatings. The scale bars for 5 nm and 50 nm AuNPs are 50 nm and 200 nm, respectively. (B) The hydrodynamic diameters and (C) zeta potentials of coated AuNPs in deionized water and DMEM with 10% FBS.

**
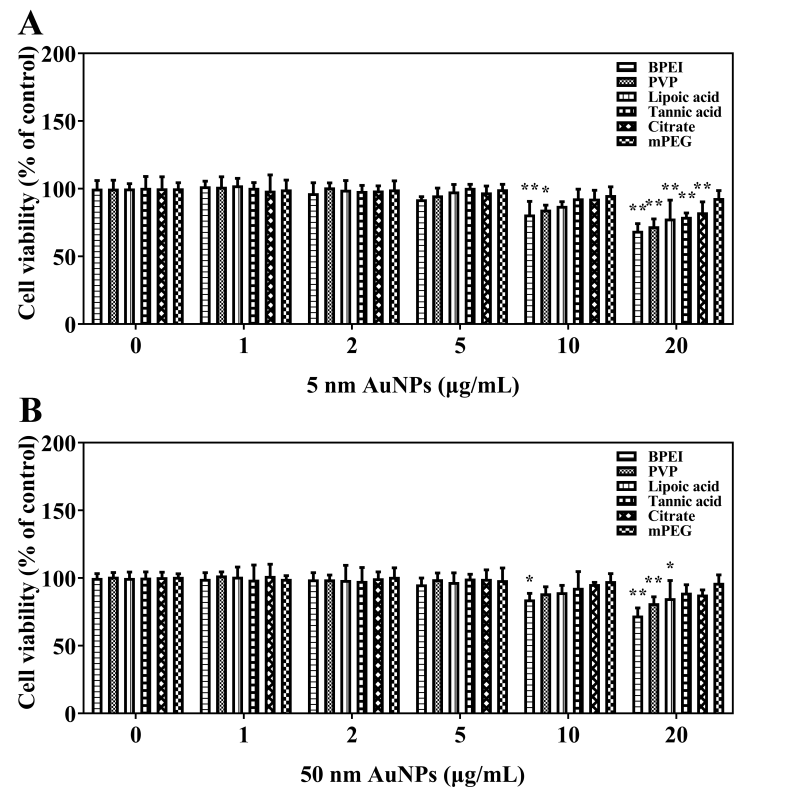
**

**Fig. S2.** Cell viability stimulated by a series of concentrations (0, 1, 2, 5, 10, and 20 μg/mL) of (A) 5 nm and (B) 50 nm AuNPs with different coatings (BPEI, PVP, lipoic acid, tannic acid, citrate, and mPEG) for 24 h. (n=3, ******p* < 0.05, *******p* < 0.01).


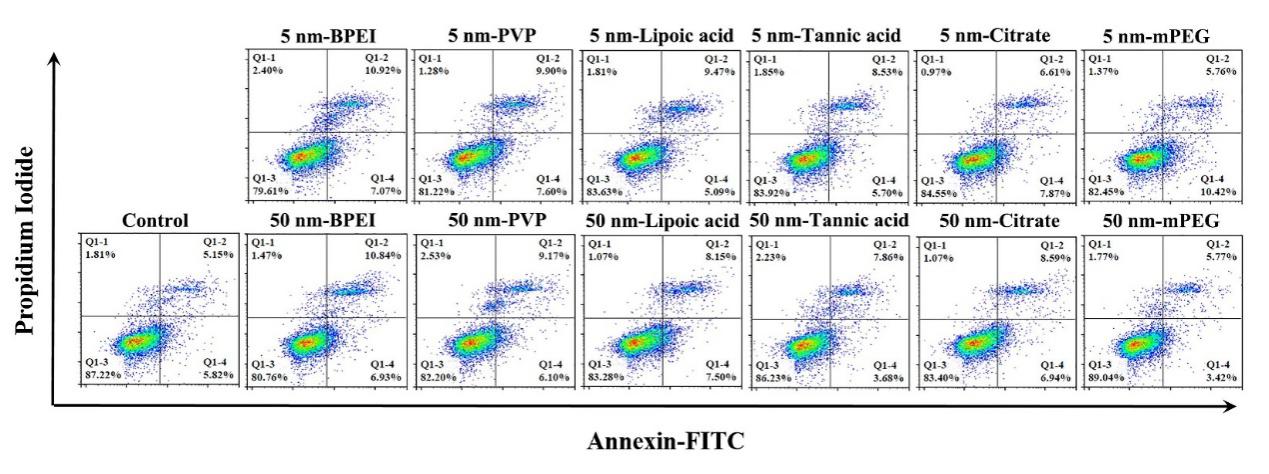


**Fig. S3.** Cell apoptosis stimulated by 5 nm and 50 nm AuNPs (5 μg/mL) with different coatings (BPEI, PVP, lipoic acid, tannic acid, citrate, and mPEG) for 24 h (n=3).


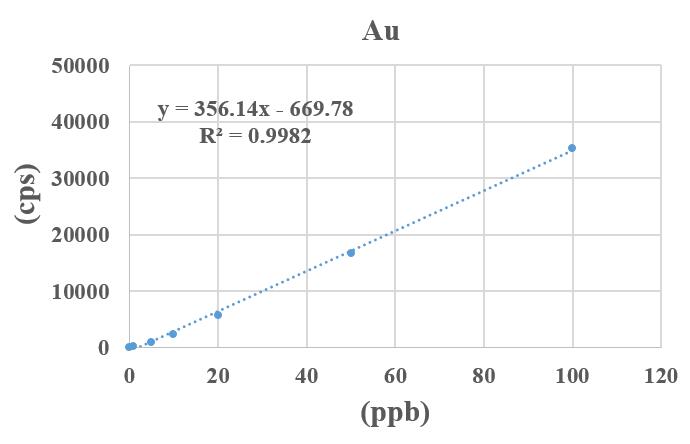


**Fig. S4.** The standard curve and regression equation of gold.


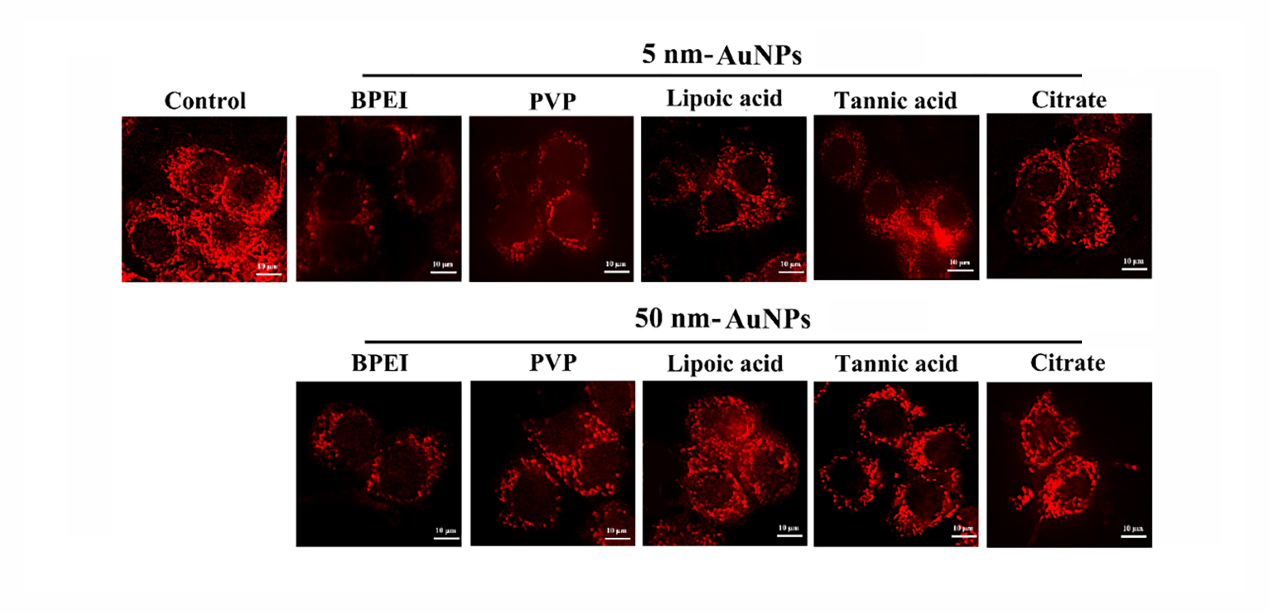


**Fig. S5.** The fluorescence images of mitochondrial morphology with Mito Tracker Red staining after different coated AuNPs (5 μg/mL) exposure for 24 h (n=3). The scale bar is 10 μm.


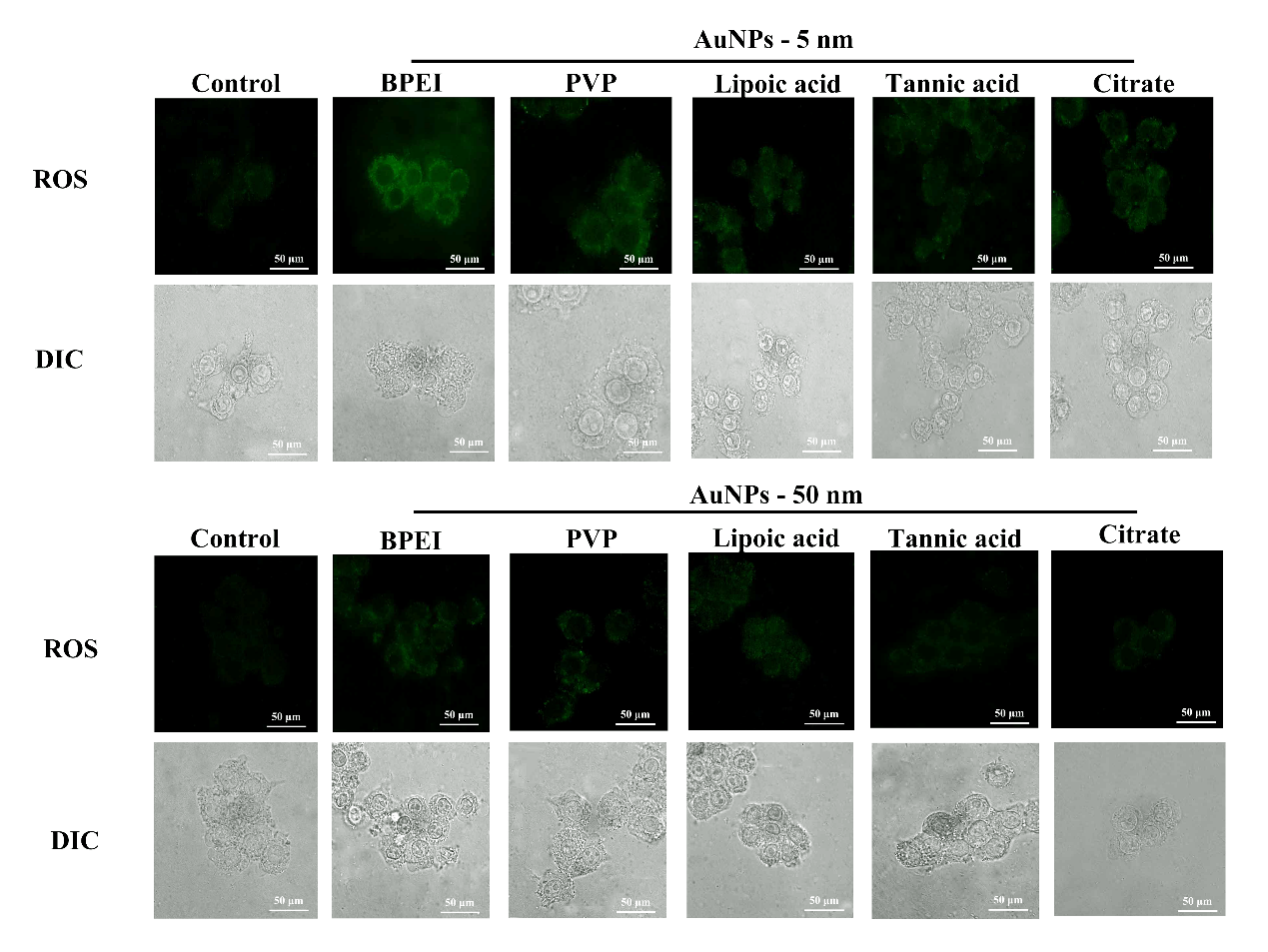


**Fig. S6.** Fluorescence images of total ROS generation after exposure of 5 nm and 50 nm different coated AuNPs (5 μg/mL) for 24 h (n=3). The scale bar is 50 μm.

**
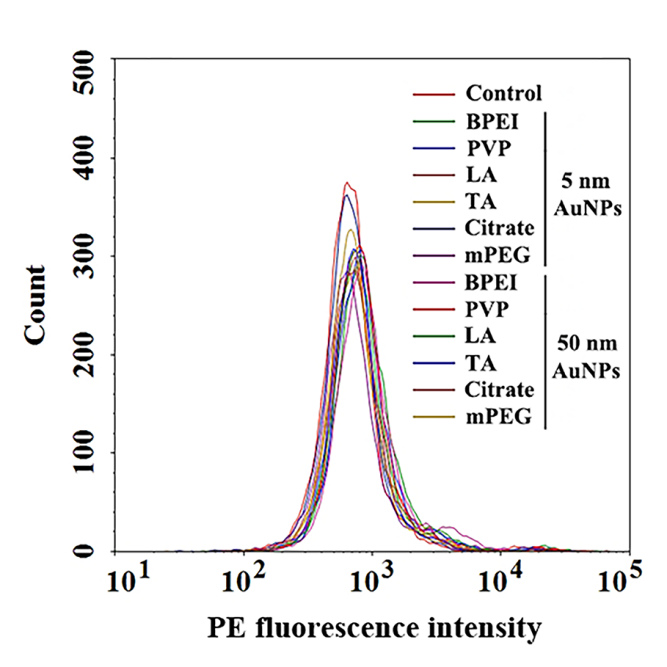
**

**Fig. S7.** The mitochondrial ROS levels in macrophages stimulated by 5 nm and 50 nm different coated AuNPs (5 μg/mL) for 24 h (n=3).


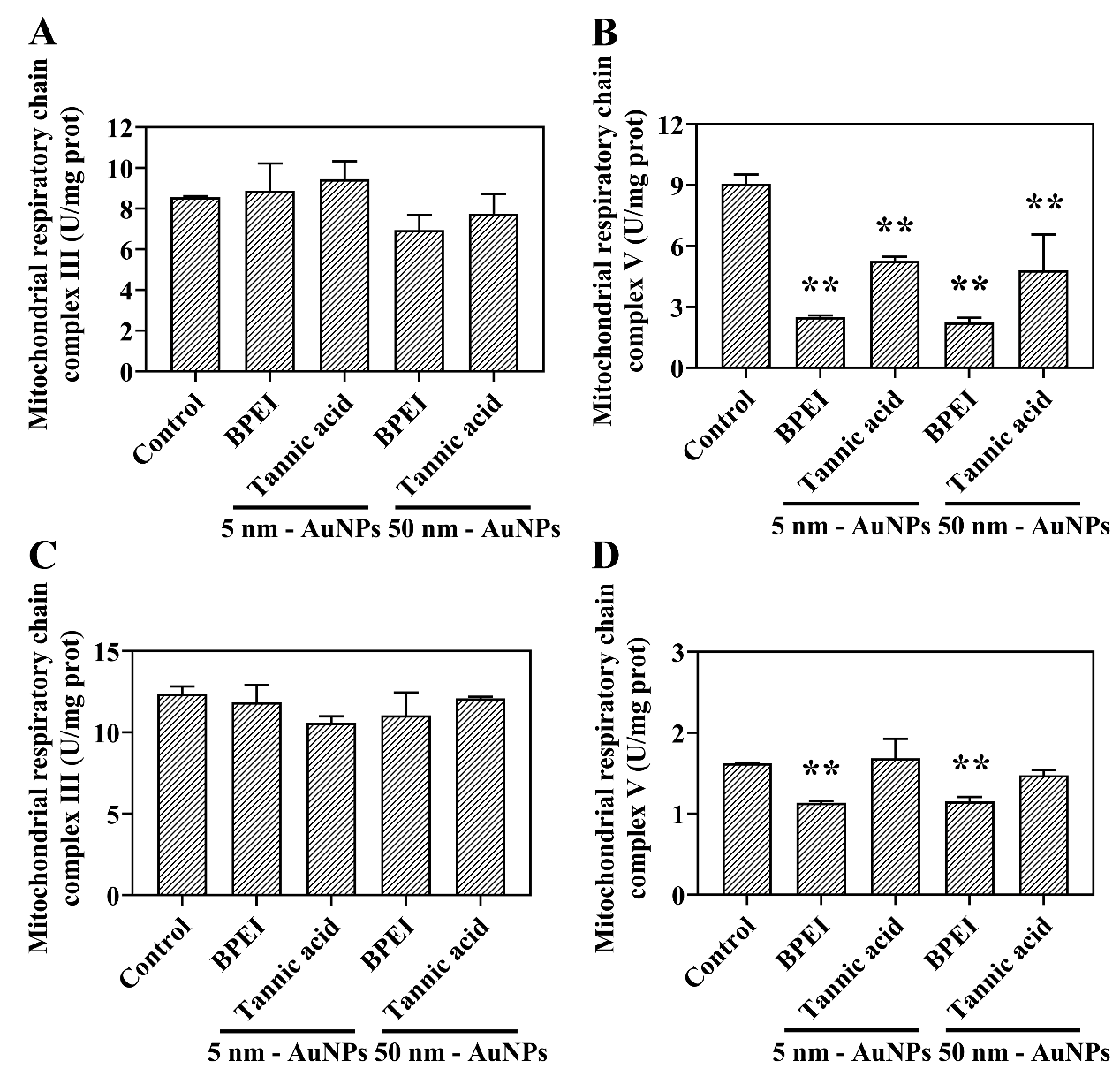


**Fig. S8.** The activities of complex Ⅲ and complex Ⅴ in macrophage upon BPEI-AuNPs and tannic acid-AuNPs treatment. The activities of (A) complex Ⅲ and (B) complex Ⅴ in RAW264.7 cells. The activities of (C) complex Ⅲ and (D) complex Ⅴ in J774A.1 cells. (n=3, ******p* < 0.05, *******p* < 0.01).


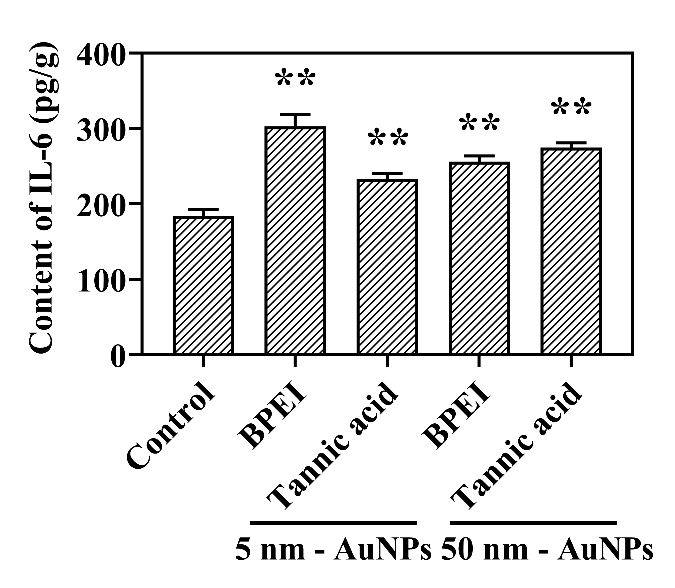


**Fig. S9.** The content of IL-6 in Raw264.7 cells treated with 5 nm and 50 nm BPEI- and tannic acid-AuNPs (5 μg/mL) for 24 h. (n=3, ******p* < 0.05, *******p* < 0.01).


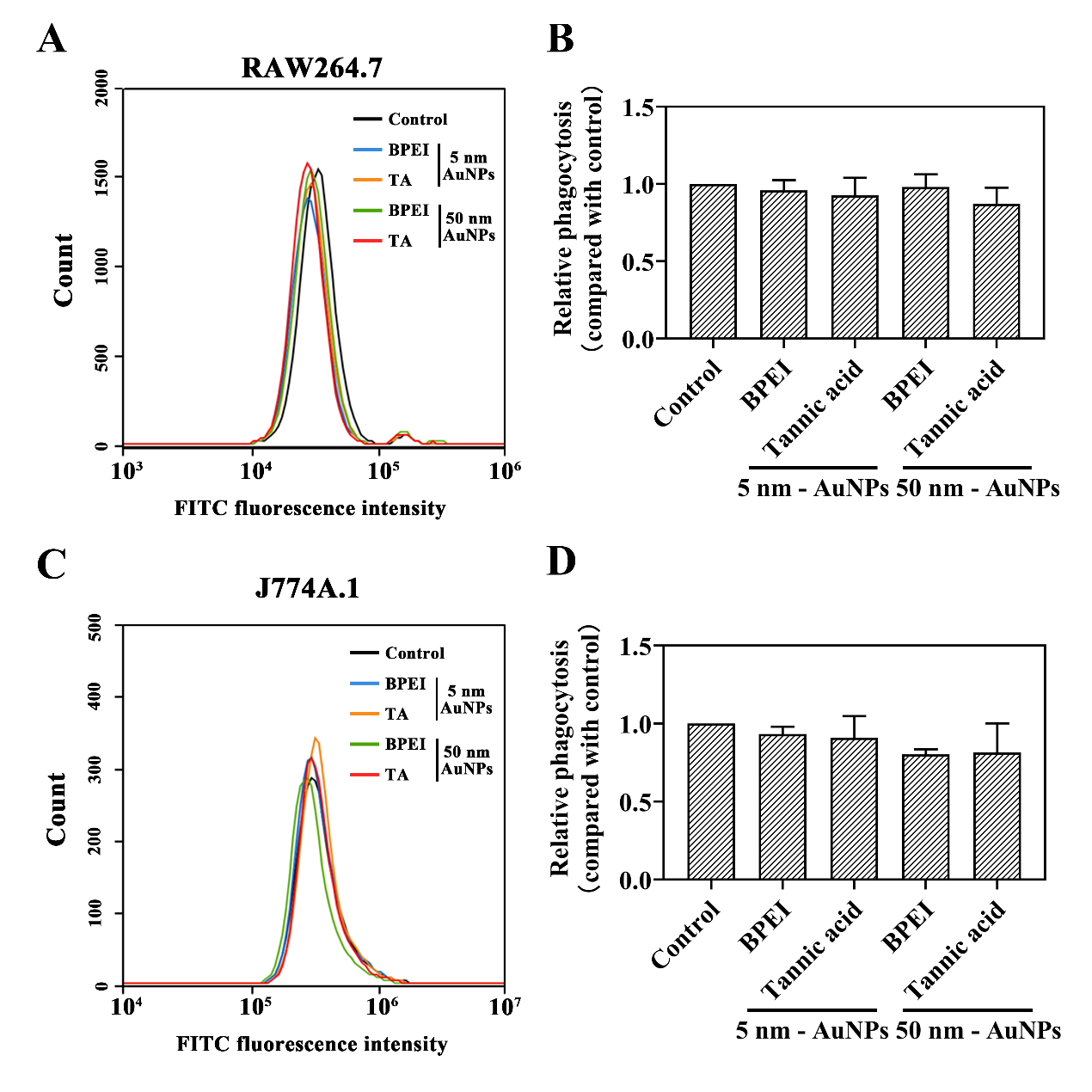


**Fig. S10.** The phagocytic capacity of macrophages after 5 nm and 50 nm BPEI- and tannic acid-AuNPs exposure. (A and B) The phagocytic function of Raw264.7 cells or (C and D) J774A.1 cells upon AuNPs treatment by flow cytometry and the quantitative result of the fluorescence intensity (n=3).


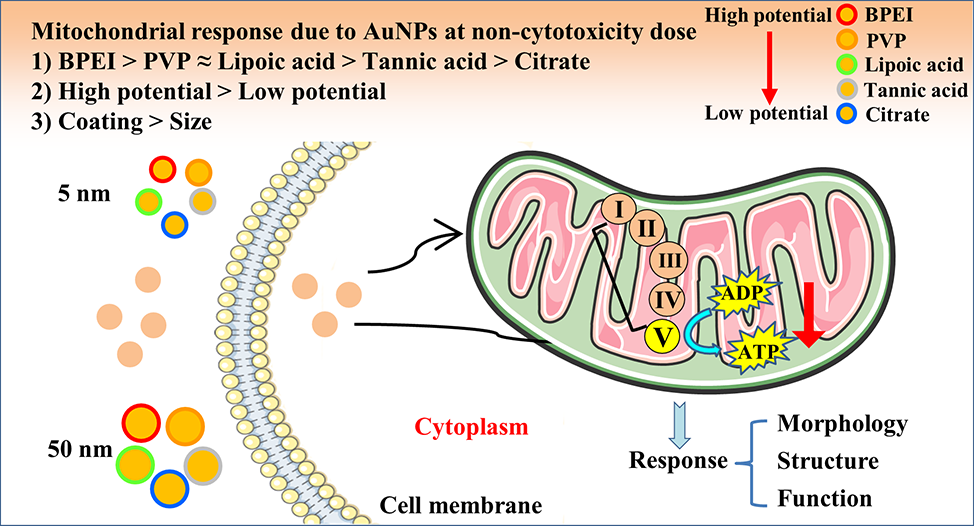


**Fig. S11.** Schematic diagram for surface charge-dependent mitochondrial response to similar intracellular nanoparticle contents at sublethal dosages.

| Group | Label | Estimate | t-Value | Probt | StandardizedEst | adj_R^2^ |
| --- | --- | --- | --- | --- | --- | --- |
| ATP | Intercept | 2.1880 | 31.1852 | 0.00E+00 | 0 | 0.8919 |
|  | 5nm | -0.2643 | -4.1272 | 2.98E-04 | -0.2514 |  |
|  | BPEI | -1.1867 | -11.3822 | 0.00E+00 | -0.8682 |  |
|  | PVP | -0.9919 | -9.5130 | 0.00E+00 | -0.7257 |  |
|  | Lipoic acid | -0.6705 | -6.4309 | 5.79E-07 | -0.4906 |  |
|  | Tannic acid | -0.4216 | -4.0441 | 3.73E-04 | -0.3085 |  |
|  | Citrate | -0.0514 | -0.4800 | 6.35E-01 | -0.0349 |  |
|  |  |  |  |  |  |  |
| Tubular mitochondria | Intercept | 108.4372 | 20.2198 | 0.00E+00 | 0 | 0.8886 |
|  | 5nm | -16.8054 | -4.9547 | 3.79E-05 | -0.3053 |  |
|  | BPEI | -81.3824 | -11.9969 | 0.00E+00 | -1.1452 |  |
|  | PVP | -71.2430 | -10.5022 | 0.00E+00 | -1.0025 |  |
|  | Lipoic acid | -51.8305 | -7.6406 | 4.10E-08 | -0.7294 |  |
|  | Tannic acid | -43.2258 | -6.3721 | 9.49E-07 | -0.6083 |  |
|  | Citrate | -35.2779 | -5.2005 | 1.98E-05 | -0.4964 |  |
|  |  |  |  |  |  |  |
| ROS | Intercept | 0.0159 | 11.4728 | 0.00E+00 | 0 | 0.805 |
|  | 5nm | 0.0085 | 4.1572 | 2.61E-04 | 0.5558 |  |
|  | 50nm | -0.0022 | -1.0592 | 2.98E-01 | -0.1416 |  |
|  | BPEI | 0.0118 | 5.9942 | 1.62E-06 | 0.5776 |  |
|  | PVP | 0.0058 | 2.9760 | 5.84E-03 | 0.2868 |  |
|  | Lipoic acid | 0.0075 | 3.8323 | 6.30E-04 | 0.3693 |  |
|  | Tannic acid | 0.0026 | 1.3168 | 1.98E-01 | 0.1269 |  |
|  |  |  |  |  |  |  |
| Fluorescence intensity | Intercept | 1.0619 | 23.2688 | 0.00E+00 | 0 | 0.8213 |
|  | 5nm | -0.1248 | -4.3232 | 2.00E-04 | -0.3375 |  |
|  | BPEI | -0.4984 | -8.6332 | 4.00E-09 | -1.0440 |  |
|  | PVP | -0.4180 | -7.2417 | 1.09E-07 | -0.8757 |  |
|  | Lipoic acid | -0.3203 | -5.5488 | 7.95E-06 | -0.6710 |  |
|  | Tannic acid | -0.2222 | -3.8494 | 6.92E-04 | -0.4655 |  |
|  | Citrate | -0.1781 | -3.0856 | 4.78E-03 | -0.3731 |  |
|  |  |  |  |  |  |  |
| Mitochondrial ROS | Intercept | 825.0000 | 21.1229 | 0.00E+00 | 0 | 0.8405 |
|  | BPEI | 533.0000 | 11.1425 | 0.00E+00 | 1.2323 |  |
|  | PVP | 397.0000 | 8.2994 | 7.00E-09 | 0.9179 |  |
|  | Lipoic acid | 300.3333 | 6.2785 | 1.02E-06 | 0.6944 |  |
|  | Tannic acid | 228.0000 | 4.7664 | 5.71E-05 | 0.5272 |  |
|  | Citrate | 163.1667 | 3.4110 | 2.05E-03 | 0.3773 |  |

**Table S1.** Multiple linear regression on the contribution of diameter and coating to the mitochondrial response.

The green tag means the *******p* < 0.01.

**Table S2.** Correlation analysis of zeta potential and different mitochondrial responses.

| Group | Medium | Variable | Estimate | Probt |
| --- | --- | --- | --- | --- |
| ATP | DMEM | Intercept | 0.567487114 | 0.000080874 |
| ATP | DMEM | Zeta potential | -0.011757 | 0.071315414 |
| ATP | Water | Intercept | 0.583506079 | 0.000004057 |
| ATP | Water | Zeta potential | -0.0050078 | 0.0147046 |
| Tubular mitochodira | DMEM | Intercept | 0.357288398 | 0.00369374 |
| Tubular mitochodira | DMEM | Zeta potential | -0.010776 | 0.113544029 |
| Tubular mitochodira | Water | Intercept | 0.371150234 | 0.000408031 |
| Tubular mitochodira | Water | Zeta potential | -0.004624 | 0.0338066 |
| ROS | DMEM | Intercept | 1.731726821 | 0.000000055 |
| ROS | DMEM | Zeta potential | 0.0204517 | 0.0272699 |
| ROS | Water | Intercept | 1.688379937 | 0.000000003 |
| ROS | Water | Zeta potential | 0.0080709 | 0.005676 |
| Mitochondrial ROS | DMEM | Intercept | 1.500389586 | 0.000000001 |
| Mitochondrial ROS | DMEM | Zeta potential | 0.0140544 | 0.0109867 |
| Mitochondrial ROS | Water | Intercept | 1.460509644 | 0 |
| Mitochondrial ROS | Water | Zeta potential | 0.00512872 | 0.00400633 |
| Fluorescence intensity | DMEM | Intercept | 0.575270017 | 0.000001305 |
| Fluorescence intensity | DMEM | Zeta potential | -0.0072319 | 0.078213335 |
| Fluorescence intensity | Water | Intercept | 0.589933783 | 0.000000088 |
| Fluorescence intensity | Water | Zeta potential | -0.0028814 | 0.0298872 |

The pink tag means the ******p* < 0.05. The green tag means the *******p* < 0.01.

**Reference**

1. Qu G, Liu S, Zhang S, Wang L, Wang X, Sun B, et al. Graphene oxide induces toll-like receptor 4 (tlr4)-dependent necrosis in macrophages. ACS Nano. 2013;7 7:5732-45. <http://dx.doi.org/10.1021/nn402330b> <https://www.ncbi.nlm.nih.gov/pubmed/23734789>.
